# Early Fesoterodine Fumarate Administration Prevents Neurogenic Detrusor Overactivity in a Spinal Cord Transected Rat Model

**DOI:** 10.1371/journal.pone.0169694

**Published:** 2017-01-06

**Authors:** Xavier Biardeau, Mikolaj Przydacz, Shachar Aharony, George Loutochin, Lysanne Campeau, Maeva Kyheng, Jacques Corcos

**Affiliations:** 1 Department of Urology, Jewish General Hospital, McGill University, Montreal, Quebec, Canada; 2 Department of Biostatistics, EA2694, University of Lille, CHRU Lille, France; University of Kentucky, UNITED STATES

## Abstract

**Background:**

In spinal cord injury, onset of detrusor overactivity (DO) is detrimental for quality of life (incontinence) and renal risk. Prevention has only been achieved with complex sophisticated electrical neuromodulation techniques.

**Purpose:**

To assess the efficacy of early fesoterodine fumarate (FF) administration in preventing bladder overactivity in a spinal cord transected (SCT) rat model.

**Methods:**

33 Sprague-Dawley rats were allocated to 6 groups–Group 1: 3 normal controls; Group 2: 6 SCT controls; Group 3: 6 SCT rats + FF 0.18 mg/kg/d; Group 4: 6 SCT rats + FF 0.12 mg/kg/d; Group 5: 6 SCT rats + FF 0.18 mg/kg/d + 72-h wash-out period; Group 6: 6 SCT rats + FF 0.12 mg/kg/d + 72-h wash-out period. SCT was performed at T10. FF was continuously administered. Cystometry was undertaken 6 weeks after SCT in awake rats recording intermicturition pressure (IMP), baseline pressure, threshold pressure (Pthres) and maximum pressure (Pmax). Normal controls and SCT controls were initially compared using the Mann-Whitney U tests in order to confirm the SCT effect on cystometric parameters. The comparisons in cystometric and metabolic cage parameters between SCT controls and treated rats were done using post-hoc Dunn’s tests for Kruskal-Wallis analysis. Statistical testing was conducted at the two-tailed α-level of 0.05.

**Results:**

Pressure parameters were significantly higher in SCT control group compared to normal controls. Six weeks after SCT, IMP was significantly lower in low dose treated group than in SCT controls. Pmax was significantly lower in 3 treated groups compared to SCT controls. Pthres was significantly lower in full time treated groups than in SCT controls.

**Conclusion:**

Early administration of FF modulates bladder overactivity in a SCT rat model. Whereas short-term prevention has been demonstrated, the long-term should be further analyzed. Clinical application of these results should confirm this finding through randomized research protocols.

## Introduction

Acute supra-sacral spinal cord injury (SCI) is initially accompanied by “spinal shock”, and then followed by emergence of a spinal-reflex-pathway after several weeks, resulting in detrusor overactivity (DO) and potential detrusor-sphincter dyssynergia. Although the treatment of DO is currently well defined and consensus-driven–with antimuscarinic drugs as the first-line treatment–its prevention has rarely been explored [1,2].

Some studies clearly support preventive strategies to avoid, delay or reduce the emergence of several pathological conditions associated with neurological diseases, such as chronic pain or spasticity [3–5]. Similarly, after acute supra-sacral SCI, early pudendal nerve stimulation (PNS) and sacral neuromodulation (SNM) have been reported to successfully obviate DO, enhancing the potential of preventive measures against SCI-related DO [6,7].

Based on current knowledge, a preventive strategy could target the muscarinic pathway. Indeed, the emergence of a spinal reflex pathway after acute SCI is responsible for significant changes in the expression of muscarinic receptor subtypes at the detrusor level, while prolonged antimuscarinic therapy has been determined to evoke opposite effects [8–10]. These results suggest that early introduction of antimuscarinic treatment could prevent SCI-related DO by antagonizing modifications potentially arising at the muscarinic pathway level.

In the present study of a spinal cord transected (SCT) rat model, we aimed to prospectively demonstrate that DO can be prevented by early fesoterodine fumarate (FF) administration introduced at the time of spinal transection to prevent bladder overactivity.

## Materials and Methods

### Ethics statement

The present research protocol (No. 2014–7459) was approved by our institutional Research Ethics Committee. All surgical procedures were performed under general anesthesia with inhaled isoflurane and analgesia with buprenorphine. The rats were euthanatized immediately after cystometry with inhaled isoflurane, O_2_ and CO_2_.

### Study design (Fig 1)

33 Sprague-Dawley rats were randomly allocated to 6 different groups:

- Group 1: 3 normal rats
- Group 2: 6 untreated SCT rats
- Group 3: 6 SCT rats + FF 0.18 mg/kg/day
- Group 4: 6 SCT rats + FF 0.12 mg/kg/day
- Group 5: 6 SCT rats + FF 0.18 mg/kg/day + 72-h wash-out period
- Group 6: 6 SCT rats + FF 0.12 mg/kg/day + 72-h wash-out period

**Fig 1:**
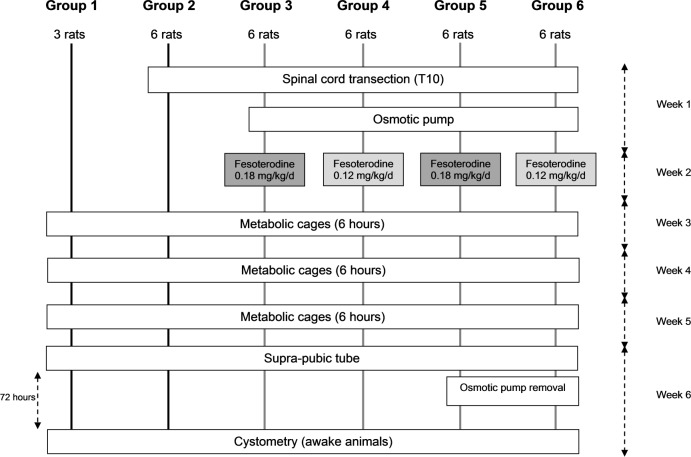
Study design.

The two distinct dosages chosen for administration in rats correspond to the drug doses usually prescribed in clinical practice to treat neurogenic DO in adult men and women– 8 mg/day and 12 mg/day (50 to 70 kg of body weight) [11–13].

Except for Group 1, all animals initially underwent SCT at T10 and were followed during 6 consecutive weeks. Metabolic cage data were recorded once a week from week 3 to 5, and cystometry was undertaken on awake animals at the end of week 6. It was performed via supra-pubic tube inserted through the bladder dome 72 h earlier. In Groups 3 to 6, FF was administered continuously by subcutaneous osmotic pump implanted at the time of SCT. The 72-h wash-out period in Groups 5 and 6 was ensured by osmotic pump removal at supra-pubic tube insertion.

### Experimental animals and housing

Female Sprague-Dawley rats, weighing 350–400 g, were delivered to our animal facilities for this study by Charles River Laboratories, Wilmington, MA, USA. Normal controls (Group 1) were housed separately in regular cages, while SCT rats (Groups 2–6) were kept separately in cages with cotton bedding and washed-dried once a day to prevent decubitus ulcers. They were examined regularly for skin blisters, and their bladder was emptied twice a day by Credé’s maneuver. The maximum time interval between 2 bladder emptyings was set at 12 h to avoid urinary retention and upper urinary tract deterioration.

### Experimental procedures

All surgical procedures were performed under general anesthesia with inhaled isoflurane for induction and maintenance. Analgesia was ensured with buprenorphine slow release (0.05 mg/kg) administered subcutaneously before SCT. Preoperative antibiotic (2.2 mg/kg trimethoprim) was injected subcutaneously just before the procedure. The animals were warmed systematically (water heated hard pads HHP-2 with Gaymar heating/cooling water pump TP-700, Braintree Scientific Inc., Braintree, MA, USA) throughout the procedure as well as in the post-operative period until full recovery (2–4 h).

### Spinalization (Groups 2–6)

Each animal was placed in a prone position and, after sterilization, its skin was incised medially at T10. A laminectomy was carried out at T10 with scissors. The spinal cord was sharply cut with Iris scissors, and a piece of Gelfoam® (Pfizer Inc., New York, NY, USA) was intercalated between the both cut ends.

### Osmotic pump implantation (Groups 3–6)

FF was administered subcutaneously by 2006 Alzet^®^ osmotic pump (Alzet Osmotic Pumps, Cupertino, CA, USA) in peri-device space–pumping rate was 0.15 μl/h, duration was 42 days, and reservoir volume was 200 μl. The pump was implanted at the time of SCT, just before skin closure, in a subcutaneous space dissected at the upper part of the neck. Osmotic pump content was calculated as FFc = K/Q, with K = Mass delivery rate (μg/h), Q = Pumping rate (μl/h), and FFc = FF concentration (μg/μl).

### Metabolic cage recording (Groups 1–6)

From weeks 3 to 5 post-SCT, individual rats were placed in metabolic cages once a week during 6 consecutive hours, with micturition volume (MVmc) and micturition frequency (MFmc) recorded by Labscribe 2^®^ software (iWorx Systems, Inc., Dover, UK). All records were performed at the same time of a day. Metabolic cage parameters were measured to portray DO emergence along the 6-week follow-up period. The bladder was emptied by Credé’s maneuver just before recording to ensure that micturitions were related to bladder contractions and not to overflow. Normal control group underwent a single metabolic cage recording before cystometry.

### Supra-pubic tube insertion

A supra-pubic tube was inserted into the bladder of all groups, 72 h before cystometry during a dedicated procedure. We followed the technique recently described by Uvin et al. [10]. The bladder was exposed through a midline incision and a P50 polyethylene tube (VWR International, Radnor, PA, USA) was inserted in the bladder dome. The tube was secured with a purse-string suture and delivered subcutaneously in the back of the animal neck.

### Osmotic pump removal (Groups 5–6)

In Groups 5 and 6, the osmotic pump was removed at the time of supra-pubic tube insertion to ensure a 72-h washout period.

### Cystometry (Groups 1–6)

Cystometry was performed on awake rats 6 weeks after SCT and 72h after supra-pubic tube insertion. Each rat was placed in a metabolic cage throughout the recording period. The bladder was initially emptied by Credé’s maneuver, and room temperature saline solution was infused at 10 cc/h. The procedure was undertaken with high-sensitivity pressure transducer (iWorx Systems Inc., Dover, NH, USA) connected to catheter and infusion pump (Harvard Apparatus, Saint Laurent, QC, Canada) via 2-way stop-cock. Intravesical pressure was monitored continuously during 3 complete voiding cycles, with Labscribe 2^®^ software (iWorx Systems Inc., Dover, NH, USA). Voided volume was recorded in parallel, with a volume transducer, to delimit voiding cycles. All cystometric parameters considered in the present study were reported according to terminology defined by Andersson et al. [14]: intermicturition pressure (IMP), maximum pressure (Pmax), baseline pressure (Pbase), threshold pressure (Pthres), micturition volume (MV), bladder capacity (BC) and residual volume (RV).

### Euthanasia

The rats were euthanasized immediately after cystometry with inhaled isoflurane and high 0_2_ concentration followed by high C0_2_ concentration.

### Experimental outcomes

IMP was considered as the primary experimental outcome. Secondary experimental outcomes were cystometric pressure and volume parameters, including Pmax, Pbase, Pthres, MV, BC and RV.

### Statistical analysis

All cystometric and metabolic cage parameters were expressed as means, medians and range. To confirm the effect of SCT on cystometric parameters, normal controls (Group 1) and SCT controls (Group 2) were initially compared using the Mann-Whitney U test. Thereafter, comparisons in cystometric and metabolic cage parameters between SCT controls (Group 2) and SCT treated rats (Groups 3–6) were done using the post-hoc Dunn’s tests for Kruskal-Wallis analysis [15]. Rats treated with FF 0.18 mg/kg/day (Group 3) and FF 0.18 mg/kg/day + wash-out period (Group 5) as well as rats treated with FF 0.12 mg/kg/day (Group 4) and FF 0.12 mg/kg/day + wash-out period (Group 6) were compared in order to assess the effect of a 72-h wash-out period on cystometric parameters, using the Mann-Whitney U test. Statistical testing was conducted at the two-tailed α-level of 0.05. Data were analyzed using the SAS software version 9.4 (SAS Institute, Cary, NC). Potential preventive effect of FF on DO emergence in acute supra-sacral SCT rats has never been reported before, rendering sample size calculation difficult from previous studies. The number of included rats was decided, in consultation with the local Research Ethics Committee, according to the principles of replacement, reduction and refinement.

## Results

**Fig 2:**
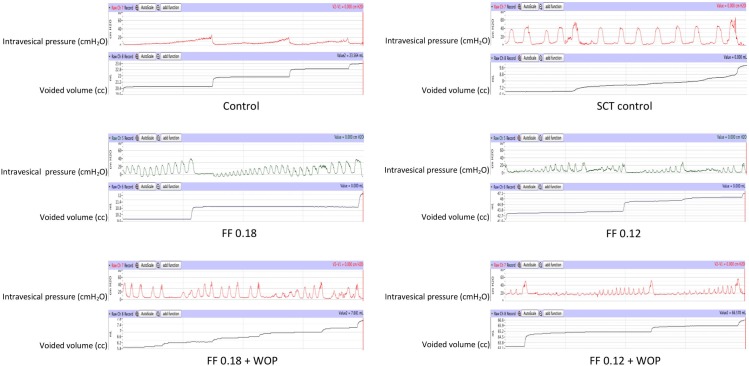
Representative cystometries, time scales used are the same in all 6 groups of animals (Groups 1–6). Control (Group 1): normal rats; SCT control (Group 2): untreated SCT rats; FF 0.18 (Group 3): SCT rats treated with FF 0.18 mg/kg/day; FF 0.12 (Group 4): SCT rats treated with FF 0.12 mg/kg/day; FF 0.18 + WOP (Group 5): SCT rats treated with FF 0.18 mg/kg/day + 72-h wash-out period; FF 0.12 + WOP (Group 6): SCT rats treated with FF 0.12 mg/kg/day + 72-h wash-out period; WOP: wash-out period.

No post-operative complications were observed in all 6 experimental groups. No mortality occurred during the entire period of the study among any of the experimental groups. Analyzed urodynamic parameters were obtained from cystometrograms with at least 3 voiding cycles (Fig 2).

### Primary experimental outcome (Fig 3)

IMP was significantly higher in SCT controls (Group 2) than in normal controls (Group 1) at the end of the 6-week follow-up period (Table 1). In contrast, 6 weeks after SCT, IMP was significantly lower in rats treated with FF 0.12 mg/kg/day (Group 4) compared to SCT controls (Group 2) (Table 2). No statistical differences were found between Groups 3 and 4 as well as between Groups 5 and 6 (Table 3).

**Fig 3:**
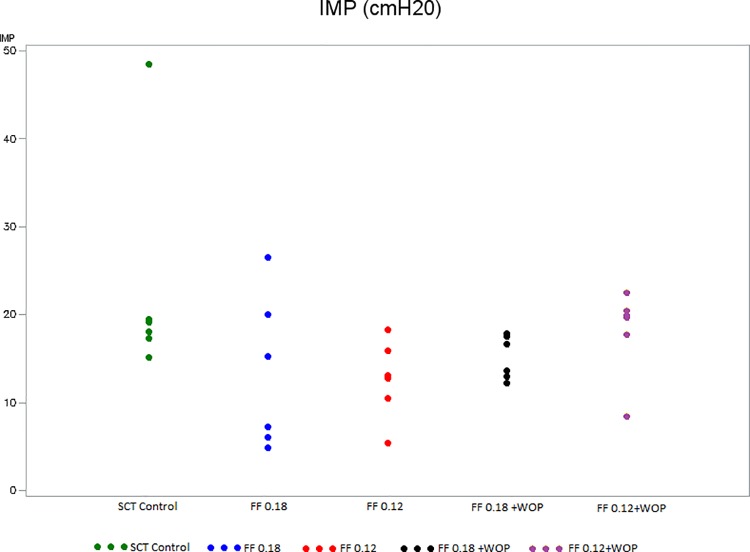
Intermicturition pressure (Groups 2–6). IMP: intermicturition pressure SCT control (Group 2): untreated SCT rats; FF 0.18 (Group 3): SCT rats treated with FF 0.18 mg/kg/day; FF 0.12 (Group 4): SCT rats treated with FF 0.12 mg/kg/day; FF 0.18 + WOP (Group 5): SCT rats treated with FF 0.18 mg/kg/day + 72-h wash-out period; FF 0.12 + WOP (Group 6): SCT rats treated with FF 0.12 mg/kg/day + 72-h wash-out period.

**Table 1:** Cystometric parameters–comparison of Group 2 to Group 1.

	Control (Group 1)	SCT control (Group 2)
**IMP**	• 6.8 • 7.2	• 22.9 • 18.6
	• (6.0–7.2)	• (15.1–48.5)
**Pmax**	• 24.0 • 26.0	• 62.6 • 61.3
	• (19.7–26.3)	• (39.2–84.5)
**Pthres**	• 13.2 • 13.7	• 35.1 • 35.6
	• (10.9–26.3)	• (23.5–48.5)
**Pbase**	• 3.6 • 3.2	• 11.8 • 9.8
	• (2.0–5.5)	• (5.2–26.3)
**MV**	• 0.9 • 0.9	• 1.5 • 1.5
	• (0.6–1.2)	• (1.2–1.6)
**BC**	• 1.1 • 1.1	• 8.4 • 8.0
	• (0.6–1.5)	• (4.8–15.6)
**RV**	• 0.2 • 0.2	• 7.2 • 6.8
	• (0.00–0.3)	• (3.6–14.1)

Values within cells are expressed in the order: mean; median; (range). IMP: intermicturition pressure; Pmax: maximum pressure; Pthres: threshold pressure; Pbase: baseline pressure; MV: micturation volume; BC: bladder capacity; RV: residual volume. Control (Group 1): normal rats; SCT control (Group 2): untreated SCT rats. All cystometric parameters were significantly different between the control group and the SCT control group using the Mann-Whitney U test.

**Table 2:** Cystometric parameters–comparison of Group 2 to Groups 3, 4, 5 and 6.

	SCT control (Group 2)	FF 0.18 (Group 3)	FF 0.12 (Group 4)	FF 0.18 + WOP (Group 5)	FF 0.12 + WOP (Group 6)
**IMP**	• 22.9 • 18.6	• 13.4 • 11.3	• 12.7* • 13.0	• 15.2 • 15.2	• 18.1 • 19.8
	• (15.1–48.5)	• (4.9–26.5)	• (5.4–18.3)	• (12.2–17.9)	• (8.4–22.5)
**Pmax**	• 62.6 • 61.3	• 39.9** • 39.7	• 37.5** • 35.9	• 40.6* • 39.3	• 43.6 • 42.8
	• (39.2–84.5)	• (34.0–45.2)	• (24.3–50.6)	• (30.0–52.0)	• (40.5–51.0)
**Pthres**	• 35.1 • 35.6	• 22.9* • 23.5	• 23.8* • 24.5	• 28.3 • 27.1	• 28.6 • 30.0
	• (23.5–48.5)	• (17.0–31.1)	• (17.0–27.7)	• (20.3–44.8)	• (17.4–38.0)
**Pbase**	• 11.8 • 9.8	• 7.7 • 5.9	• 8.5 • 9.5	• 8.8 • 9.5	• 11.8 • 13.3
	• (5.2–26.3)	• (3.0–18.1)	• (2.0–11.5)	• (5.2–12.7)	• (7.2–15.0)
**MV**	• 1.5 • 1.5	• 1.2 • 0.6	• 1.3 • 1.3	• 1.2 • 1.1	• 1.0 • 1.0
	• (1.2–1.6)	• (0.5–2.0)	• (0.6–2.1)	• (0.5–2.1)	• (0.2–1.7)
**BC**	• 8.4 • 8.0	• c5.2 • 4.7	• 7.1 • 7.3	• 5.3 • 6.0	• 6.1 • 6.0
	• (4.8–15.6)	• (2.5–10.9)	• (2.5–11.6)	• (2.0–7.6)	• (3.8–10.1)
**RV**	• 7.2 • 6.8	• 4.1 • 4.1	• 5.9 • 5.6	• 4.1 • 5.1	• 5.2 • 5.0
	• (3.6–14.1)	• (0.5–9.4)	• (2.0–10.2)	• (0.9–6.5)	• (2.9–8.4)

Values within cells are expressed in the order: mean; median; (range). IMP: intermicturition pressure; Pmax: maximum pressure; Pthres: threshold pressure; Pbase: baseline pressure; MV: micturation volume; BC: bladder capacity; RV: residual volume; WOP: wash-out period. SCT control (Group 2): untreated SCT rats; FF 0.18 (Group 3): SCT rats treated with FF 0.18 mg/kg/day; FF 0.12 (Group 4): SCT rats treated with FF 0.12 mg/kg/day; FF 0.18 + WOP (Group 5): SCT rats treated with FF 0.18 mg/kg/day + 72-h wash-out period; FF 0.12 + WOP (Group 6): SCT rats treated with FF 0.12 mg/kg/day + 72-h wash-out period.

*: p<0.05 compared to SCT controls (Group 2) using the Post-hoc Dunn’s test for Kruskal-Wallis analysis.

**: p<0.01 compared to SCT controls (Group 2) using the Post-hoc Dunn’s test for Kruskal-Wallis analysis.

**Table 3:** Cystometric parameters–comparison of Group 3 to Group 5 and Group 4 to Group 6.

	FF 0.18 (Group 3)	FF 0.18 + WOP (Group 5)	FF 0.12 (Group 4)	FF 0.12 + WOP (Group 6)
**IMP**	• 13.4 • 11.3	• 15.2 • 15.2	• 12.7 • 13.0	• 18.1 • 19.8
	• (4.9–26.5)	• (12.2–17.9)	• (5.4–18.3)	• (8.4–22.5)
**Pmax**	• 39.9 • 39.7	• 40.6 • 39.3	• 37.5 • 35.9	• 43.6 • 42.8
	• (34.0–45.2)	• (30.0–52.0)	• (24.3–50.6)	• (40.5–51.0)
**Pthres**	• 22.9 • 23.5	• 28.3 • 27.1	• 23.8 • 24.5	• 28.6 • 30.0
	• (17.0–31.1)	• (20.3–44.8)	• (17.0–27.7)	• (17.4–38.0)
**Pbase**	• 7.7 • 5.9	• 8.8 • 9.5	• 8.5 • 9.5	• 11.8 • 13.3
	• (3.0–18.1)	• (5.2–12.7)	• (2.0–11.5)	• (7.2–15.0)
**MV**	• 1.2 • 0.6	• 1.2 • 1.1	• 1.3 • 1.3	• 1.0 • 1.0
	• (0.5–2.0)	• (0.5–2.1)	• (0.6–2.1)	• (0.2–1.7)
**BC**	• 5.2 • 4.7	• 5.3 • 6.0	• 7.1 • 7.3	• 6.1 • 6.0
	• (2.5–10.9)	• (2.0–7.6)	• (2.5–11.6)	• (3.8–10.1)
**RV**	• 4.1 • 4.1	• 4.1 • 5.1	• 5.9 • 5.6	• 5.2 • 5.0
	• (0.5–9.4)	• (0.9–6.5)	• (2.0–10.2)	• (2.9–8.4)

Values within cells are expressed in the order: mean; median; (range). IMP: intermicturition pressure; Pmax: maximum pressure; Pthres: threshold pressure; Pbase: baseline pressure; MV: micturation volume; BC: bladder capacity; RV: residual volume; WOP: wash-out period. FF 0.18 (Group 3): SCT rats treated with FF 0.18 mg/kg/day; FF 0.18 + WOP (Group 5): SCT rats treated with FF 0.18 mg/kg/day + 72-h wash-out period; FF 0.12 (Group 4): SCT rats treated with FF 0.12 mg/kg/day; FF 0.12 + WOP (Group 6): SCT rats treated with FF 0.12 mg/kg/day + 72-h wash-out period. No statistical difference was noted between rats treated with FF 0.18 (Group 3) and FF 0.18 + WOP (Group 5) as well as between rats treated with FF 0.12 (Group 4) and FF 0.12 + WOP (Group 6) using the Post-hoc Dunn’s test for Kruskal-Wallis analysis.

### Maximum pressure (Fig 4)

Pmax was significantly higher in SCT controls (Group 2) than in normal controls (Group 1) at the end of the 6-week follow-up period (Table 1). In contrast, 6 weeks after SCT, Pmax was significantly lower in most of the SCT treated rats (Groups 3–5) compared to SCT controls (Group 2) (Table 2). No statistical differences were apparent between Groups 3 and 5 as well as between Groups 4 and 6 (Table 3).

**Fig 4:**
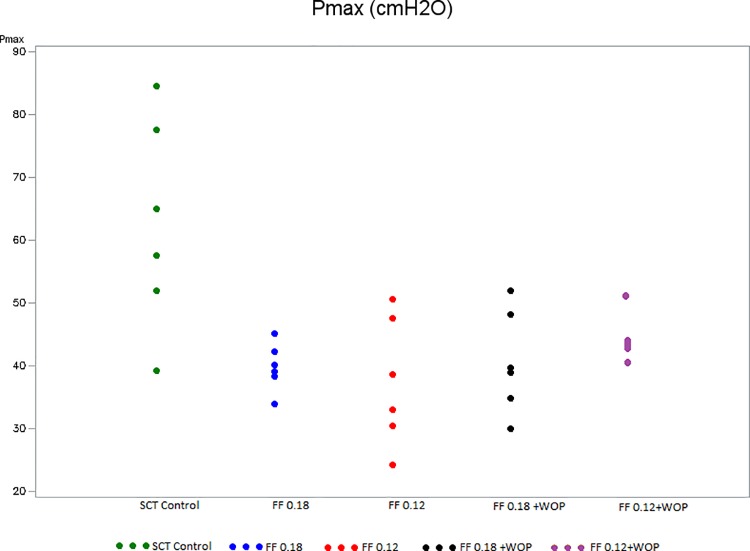
Maximum pressure (Groups 2–6). Pmax: Maximum pressure SCT control (Group 2): untreated SCT rats; FF 0.18 (Group 3): SCT rats treated with FF 0.18 mg/kg/day; FF 0.12 (Group 4): SCT rats treated with FF 0.12 mg/kg/day; FF 0.18 + WOP (Group 5): SCT rats treated with FF 0.18 mg/kg/day + 72-h wash-out period; FF 0.12 + WOP (Group 6): SCT rats treated with FF 0.12 mg/kg/day + 72-h wash-out period.

#### Threshold pressure (Fig 5)

Pthres was significantly higher in SCT controls (Group 2) than in normal controls (Group 1) at the end of the 6-week follow-up period (Table 1). In contrast, 6 weeks after SCT, Pthres was significantly lower in full time treated rats (Group 3–4) compared to SCT controls (Group 2) (Table 2). No statistical differences were noted between Groups 3 and Group 5 as well as between Groups 4 and 6 (Table 3).

**Fig 5:**
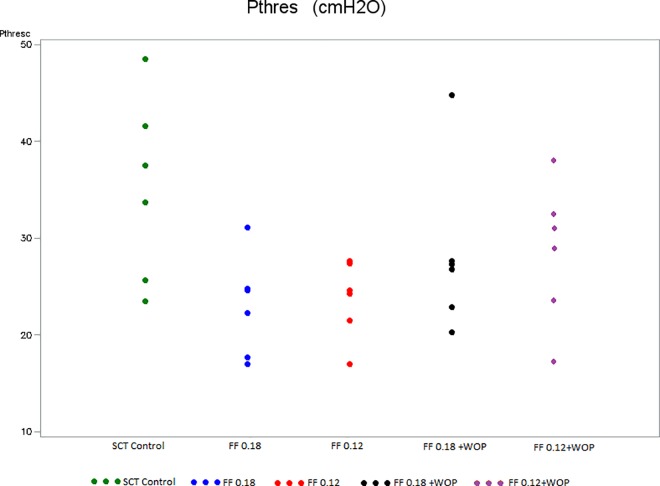
Threshold pressure (Groups 1–6). Pthres: Threshold pressure SCT control (Group 2): untreated SCT rats; FF 0.18 (Group 3): SCT rats treated with FF 0.18 mg/kg/day; FF 0.12 (Group 4): SCT rats treated with FF 0.12 mg/kg/day; FF 0.18 + WOP (Group 5): SCT rats treated with FF 0.18 mg/kg/day + 72-h wash-out period; FF 0.12 + WOP (Group 6): SCT rats treated with FF 0.12 mg/kg/day + 72-h wash-out period.

### Other cystometric parameters

Pbase, MV, BC and RV were significantly different in SCT controls (Group 2) than in normal controls (Group 1) at the end of the 6-week follow-up period, while none of these parameters was seen to be statistically different in SCT treated rats (Groups 3–6) respectively compared to SCT controls (Group 2) (Table 1).

### Metabolic cage parameters

Metabolic cage parameters (MVmc and MFmc) were not significantly different in SCT rats (Groups 2 to 6). All SCT rats (Groups 2 to 6) went through an initial period of urinary retention after SCT and presented with micturitions at the time of first metabolic cage recording, at week 3, consistent with emergence of bladder overactivity.

## Discussion

Here, we report results regarding short-term bladder overactivity prevention after acute supra-sacral SCI using early FF administration. We have shown that cystometric pressure parameters, including IMP, Pmax and Pthres, were lowered in SCT rats treated with FF compared to untreated SCT controls, persistent after cessation of treatment. According to Andersson et al. [14], IMP is highly correlated with DO and, therefore, its significant reduction with fesoterodine treatment after acute supra-sacral SCI argues for DO prevention after SCI. Interestingly, pressure parameters, measured in both 72-h wash-out period groups (Groups 5 and 6), were not significantly different compared to those in the treated groups without wash-out (Groups 3 and 4). It seems then possible, that early and chronic FF administration could decrease pressure parameters not only through an acute pharmacological effect, but also by countering pathological modifications of muscarinic pathways.

Early interventions to avoid DO associated with SCI have already been described by a few authors, using PNS or bilateral SNM. Li et al. [6] compared urodynamic parameters measured at 1 and 3 months after T9-T10 surgical SCT in 2 distinct groups of beagle dogs. One group of 3 SCT dogs was subjected to early low-frequency PNS, while the second group of 3 SCT was controls. The authors observed an inhibitory effect of early PNS and a significant decrease in non-voiding contractions coupled with the maintenance of normal bladder capacity and compliance in treated SCT dogs. Furthermore, histological analysis demonstrated significant differences in terms of collagen and elastic fiber proportions, with bladder fibrosis being lower in treated than in untreated dogs. They concluded that low-frequency PNS, early after acute supra-sacral SCI, could inhibit DO, increase bladder capacity, and delay bladder fibrosis, maintaining compliance.

Sievert et al. [7] reported similar results with bilateral SNM in humans. They compared a group of 10 complete SCT patients (T2-T11), subjected to bilateral SNM during the “spinal shock” phase, with a control group of 6 complete SCT patients. Videourodynamics study and electromyography were performed at 3 and 6 months as well as every 6 months thereafter. Mean follow-up of 26.2 (5.4–38.9) months disclosed decreased bladder pressures and increased bladder capacity in patients with early bilateral SNM compared to untreated controls. They concluded that early SNM could prevent DO after acute supra-sacral SCI.

Although antimuscarinic drugs have not yet been studied under such a short-term preventive strategy, we think that antagonizing and possibly modulating the muscarinic pathway early after acute supra-sacral SCI could represent an interesting and easy approach to DO preclusion.

In the normal bladder, the detrusor muscle mainly contains M2 and M3 muscarinic receptors, with the M3 subtype playing a major role in detrusor contraction [16]. Interestingly, the proportion and role of these detrusor muscarinic receptors have recently been demonstrated to evolve in opposite directions after acute supra-sacral SCI and chronic antimuscarinic treatment, respectively. According to Somogyi et al. [9] post-ganglionic parasympathetic nerve terminals become more sensitive to the facilitatory action of endogenously released acetylcholine and enhance transmitter release at lower frequencies of nerve stimulation and at lower acetylcholine concentrations.

Similarly, Braverman et al. [8] compared the density of total muscarinic receptors as well as the density of M2 and M3 receptor subtypes at the detrusor level in SCI rats (10 days after T9 spinal cord compression) and controls. They reported that total muscarinic receptor density was significantly higher in SCI rats than in normal controls. M2 receptors accounted for the entire increase, with no change in M3 receptor density. Furthermore, they demonstrated a switch from M3-mediated detrusor contractions in normal rats to M2-mediated detrusor contractions in SCI rats.

In contrast, Uvin et al. [10] studied the effect of prolonged antimuscarinic therapy at the detrusor level in normal rats and noted down regulation of muscarinic postsynaptic responses, including decreased M2 and M3 muscarinic receptor expression. These results clearly underline significant modifications emerging at the muscarinic pathway level after acute supra-sacral SCI with opposite changes occurring after chronic anticholinergic drug administration, making them good candidates for DO prevention.

Interpretation of the present findings is, however, limited by the absence of pathological and immunohistochemical analysis of bladder samples. Furthermore, it would have been ideal to perform a 24-hour metabolic cage recording for each animal. However, due to the loss of rats sensitivity and the emergence of involuntary movements of lower limbs animals were prone to legs injuries and skin damages and required to be continuously monitored during the whole recordings. We also acknowledge that IMP, Ptres and Pmax characterize voiding in rodents and not in humans. Nevertheless, they can be used as proxy for certain cystometric variables in patients as stated by Andersson et al. [14].

## Conclusion

Early FF administration at the time of transection modulates bladder overactivity. Whereas short-term prevention has been demonstrated, the long-term should be further analyzed. The exact mechanism of action should be investigated through further pathological and immunohistochemical studies focusing on detrusor muscarinic receptor modifications from preventive therapy. The obtained results may support clinical trials for neurogenic bladder prevention with anticholinergics in patients after spinal cord injury.

## Abbreviations

BC: bladder capacity during cystometry
DO: detrusor overactivity
FF: fesoterodine fumarate
IMP: intermicturition pressure
MFmc: micturition frequency during metabolic cage recording
MV: micturition volume during cystometry
MVmc: micturition volume during metabolic cage recording
Pbase: baseline pressure
Pmax: maximum pressure
PNS: pudendal nerve stimulation
Pthres: threshold pressure
RV: residual volume during cystometry
SCI: spinal cord injury
SCT: spinal cord transection
SNM: sacral neuromodulation
WOP: wash-out period
